# A Two-Stage Unet Framework for Sub-Resolution Assist Feature Prediction

**DOI:** 10.3390/mi16111301

**Published:** 2025-11-20

**Authors:** Mu Lin, Le Ma, Lisong Dong, Xu Ma

**Affiliations:** 1 Key Laboratory of Photoelectronic Imaging Technology and System of Ministry of Education of China, School of Optics and Photonics, Beijing Institute of Technology, Beijing 100081, China; 2 State Key Laboratory of Fabrication Technologies for Integrated Circuits, Beijing 100029, China; 3 Institute of Microelectronics of the Chinese Academy of Sciences, Beijing 100029, China

**Keywords:** sub-resolution assist feature, two-stage Unet, adaptive hybrid attention mechanism, warm-up cosine annealing algorithm

## Abstract

Sub-resolution assist feature (SRAF) is a widely used resolution enhancement technology for improving image contrast and the common process window in advanced lithography processes. However, both model-based SRAF and rule-based SRAF methods suffer from challenges of adaptability or high computational cost. The primary learning-based SRAF method adopts an end-to-end mode, treating the entire mask pattern as a pixel map, and it is difficult to obtain precise geometric parameters for the commonly used Manhattan SRAFs. This paper proposes a two-stage Unet framework to effectively predict the centroid coordinates and dimensions of SRAF polygons. Furthermore, an adaptive hybrid attention mechanism is introduced to dynamically integrate global and local features, thus enhancing the prediction accuracy. Additionally, a warm-up cosine annealing learning rate strategy is adopted to improve the training stability and convergence speed. Simulation results demonstrate that the proposed method accurately and rapidly estimates the SRAF parameters. Compared to traditional neural networks, the proposed method can better predict SRAF patterns, with the mean pattern error and edge placement error values showing the most significant reductions. PE decreases from 25,776.44 to 15,203.33 and EPE from 5.8367 to 3.5283, respectively. This significantly improves the image fidelity of the lithography system.

## 1. Introduction

As lithography technology nodes continue to shrink, the critical dimensions (CDs) of integrated circuits have approached or surpassed the resolution limit of the optical lithography system, thus leading to significant patterning distortion in wafer images due to the optical proximity effect. A major challenge to maintaining lithography image fidelity is that the process window (PW) for isolated patterns is misaligned with that for dense patterns, thereby compromising the common PW across the whole layout. To expand the PW for isolated patterns, sub-resolution assist features (SRAFs) are placed around the main patterns of the mask [1]. This approach changes the environments of isolated patterns to effectively simulate the imaging characteristics of dense patterns, thereby extending their common PWs. Hereafter, the SRAFs refer to the non-printable tiny features inserted surrounding the main patterns on the mask. Those features modulate the local optical interference to improve the imaging contrast, depth of focus, and common PWs, thereby ensuring preferable image fidelity and manufacturability.

SRAF insertion techniques are primarily divided into the rule-based SRAF (RB-SRAF) methods [2,3,4,5,6] and model-based SRAF (MB-SRAF) methods [7,8,9,10,11]. RB-SRAF relies on predefined geometric rules and lookup tables to generate SRAFs. However, its effectiveness is inherently constrained by the completeness of the rule tables. Meanwhile, MB-SRAF employs lithography simulation models or inverse-optimization algorithms to adaptively generate SRAFs. However, as the CD of integrated circuits continues to shrink, the computational complexity of MB-SRAF increases dramatically.

In recent years, the rapid development of machine learning and deep learning has offered a promising alternative for SRAF insertion [12,13,14]. Based on data-driven strategies, researchers reformulated the SRAF insertion as an image-processing problem, using learning-based models or deep neural networks to directly predict the optimal SRAF deployment. For example, Ciou et al. used the cycle generative adversarial network (Cycle-GAN) to enhance the efficiency of SRAF insertion [15]. Wang et al. applied the deep convolutional network combined with continuous transmission masks (CTMs) to implement the rapid full-chip SRAF insertion [16]. Mohamed employed the conditional generative adversarial network (CGAN) to generate SRAFs [17]. Those methods mainly predict SRAFs with freeform shapes or curvilinear patterns, but the increasingly complex and large-scale nature of SRAF patterns poses a significant challenge to manufacturability [18,19]. In order to reduce mask complexity, some methods were proposed to simplify curvilinear SRAFs into rectangular ones to balance manufacturability and image fidelity [20,21]. However, the geometric transformation and reshape processes will undoubtedly introduce additional runtime and image fidelity loss.

**Figure 1:**
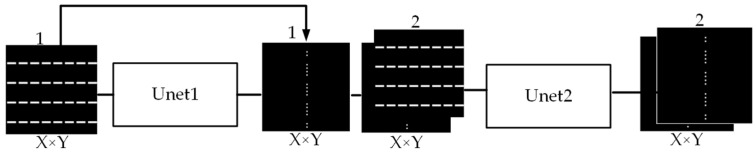
The structure of the proposed two-stage Unet framework.

In order to overcome these limitations, this paper proposes a two-stage Unet architecture to directly generate preferred SRAFs with rectangular shapes, as illustrated in Figure 1. In the first stage, the sub-network “Unet1” is used to predict the centroid coordinates of SRAFs. Given the input layout with feature size of X×Y and channel count of 1, this network uses an encoder–decoder structure to extract both global features and local details of the layout pattern. With skip connections, the network effectively preserves the high-resolution feature map and outputs the map of centroid coordinates. In the second stage, the sub-network “Unet2” with channel count of 2 predicts the dimension of each SRAF polygon. Under the constraints imposed by the centroid coordinates, it establishes the mapping relationship from key parameters, such as mask pattern shapes, densities, and spacings between main features, to the lateral sizes (height and width) of SRAF polygons. By combining the outputs of Unet1 and Unet2, the rectangular SRAFs can be inserted on the mask.

Additionally, we introduce an adaptive hybrid attention mechanism (AHAM) into the Unet architecture. This mechanism dynamically adjusts the weights between global and local information in the mask feature map to enhance the network’s ability to capture the critical features and improve the prediction accuracy. Moreover, a warm-up cosine annealing (WCA) algorithm is introduced during network training to improve the training stability and the final prediction performance.

The remainder of this paper is organized as follows. Section 2 describes the Unet structure, AHAM, and WCA algorithm. Section 3 presents the two-stage network architecture proposed in this work and details its operational workflow. Section 4 provides the numerical experimental results. Section 5 is the conclusion.

## 2. Preliminaries

### 2.1. Unet

**Figure 2:**
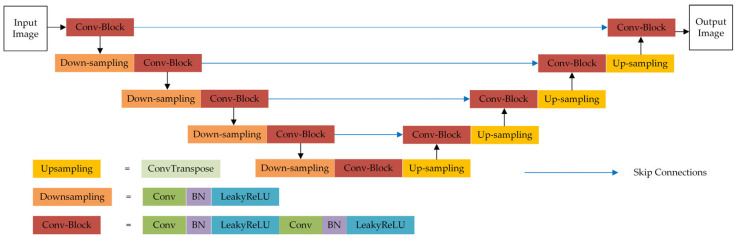
The structure of the Unet framework.

In this paper, the SRAF prediction model is constructed based on the Unet framework. As illustrated in Figure 2, Unet has unique encoder–decoder structure and cross-layer feature fusion mechanism [22], which are beneficial to mask parameter prediction directly from the layout pattern. Unet employs a U-shaped symmetric structure. The left-side encoder path performs the down-sampling operations using 3×3 convolutional kernels with a stride of 2. Combined with the LeakyReLU activation functions, the encoder hierarchically extracts the high-level features including the geometric and spatial information from the layout pattern. The right-side decoder path gradually reconstructs the resolution of feature maps through the transposed convolutions (ConvTranspose in Figure 2).

A key advantage of Unet lies in its skip connections, which concatenate feature maps from the decoder and encoder paths at the same level. This design effectively integrates high-level abstract features with low-level detailed features. Specifically, the low-resolution feature maps containing high-level semantic information are up-sampled in the decoder and then fused with the high-resolution feature maps with detailed context from the encoder via channel-wise concatenation. This fusion method enables the network to utilize feature information on different scales and enhances the model’s understanding of layout details and global semantics. Finally, the network further processes and integrates the fused feature maps to generate the final output.

### 2.2. AHAM

In deep learning, attention mechanisms allocate weights to emphasize the crucial information, thereby significantly enhancing the learning capacity of networks. Existing research demonstrates that global attention mechanisms can effectively capture the holistic semantic information by establishing long-range dependency [23], while local attention mechanisms concentrate on the localized map features, exhibiting notable advantages in preserving the detailed information [24]. These two mechanisms are complementary, but the synergy between them in feature extraction is insufficient [25,26,27].

**Figure 3:**
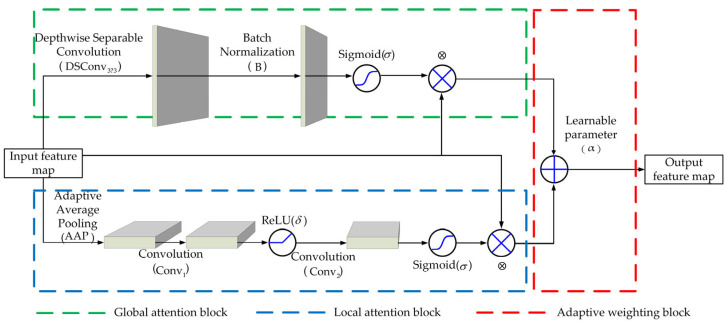
The structure of the AHAM module.

To address this issue, we propose the AHAM module, and its innovation is manifested in two aspects. First, it employs a parallel architecture with dual branches to model the global context and extract local detailed features simultaneously. Second, it introduces learnable weighting parameters to dynamically fuse the global and local attention branches, thereby enhancing the capability to extract the layout features. The structure of AHAM is shown in Figure 3.

The AHAM consists of three components, including a global attention block (green dashed line), a local attention block (blue dashed line), and an adaptive weighting block (red dashed line). The local attention block captures the structural details within the regional receptive fields, which is formulated as follows:

(1) $$F_{local}(x)=\sigma \{B[DSConv_{3\times 3}(x)]\}⊗x,$$

where x denotes the input feature map, and $DSConv_{3\times 3}$ denotes a depth-wise separable convolution with a 3×3 kernel. This operation decouples the computational flow across channels, effectively reducing the parameter count while maintaining the key local features. The notation B represents the batch normalization layer. The sigmoid activation function σ projects the output value into the range of (0,1), generating a local attention weight map with the same size as the input feature map. The weight map is then fused with the original input by element-wise multiplication ⊗ to enhance local feature representation.

The global attention block captures the semantic information of the feature map, and can be formulated as follows:

(2) $$F_{global}(x)=\sigma [Conv_{2}(\delta \{Conv_{1}[AAP(x)]\})]⊗x,$$

where AAP denotes the adaptive average pooling operation, which compresses the input feature map into a global feature vector, providing a compact representation of the global context. The $Conv_{1}$ and $Conv_{2}$ represent the convolutional layers, and δ is a nonlinear activation function that enhances the representation capacity. The resulting output is passed through the sigmoid function to generate the global attention map.

Furthermore, a learnable parameter a, whose value is dynamically adjusted by the back-propagation algorithm, is used to balance the contributions of the global and local attention branches and enable the adaptive feature integration. Thus, the final output is given by the following:

(3) $$y=\alpha \cdot F_{local}+(1-\alpha )\cdot F_{global}.$$

### 2.3. WCA Algorithm

The strategy for updating the learning rate influences the training stability, convergence speed, and generalization capacity. Although constant learning rates and piecewise constant decay strategies are widely used, their limitations have been proven in many studies [28,29]. The former one maintains an unchanged rate, which lacks flexibility for the training process, particularly in large-scale models. The latter sets different learning rates in stages. However, some critical hyperparameters, e.g., the decay step and decay factor, require tuning through numerous training and validation processes, which demands substantial computational resources.

To improve the training stability and convergence, we adopt the WCA algorithm, combining the linear warm-up [30] and cosine annealing [31], to adjust the learning rate. This method starts from the warm-up stage, stabilizes the early training process by gradually increasing the learning rate, and then enters the cosine annealing stage, where the learning rate is smoothly reduced to ensure the final convergence. During the warm-up stage, the learning rate increases linearly from 0 to a preset maximum, allowing the optimizer to initially explore the effective update directions and mitigate early instability due to the gradient anomalies or parameter randomness [30]. The warm-up phase is formulated as follows:

(4) $$lr=lr_{0}\times \frac{E}{E_{warm\text{-}up}},$$

where lr represents the current learning rate, $lr_{0}$ denotes the preset maximum rate, E is the current epoch, and $E_{warm\text{-}up}$ denotes the total number of warm-up epochs.

**Figure 4:**
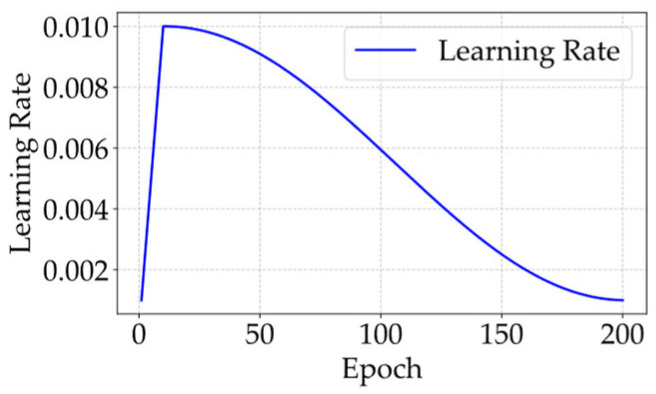
The change in learning rate with respect to the epoch number according to the WCA algorithm.

After the warm-up, the cosine annealing is used to decay the learning rate from $lr_{0}$ to the minimum based on the cosine function, which is formulated as follows:

(5) $$lr=lr_{0}\times \{min\_lr\_ratio+(1-min\_lr\_ratio)\times 0.5\times [1+cos(\pi \times \frac{E-E_{warm-up}}{E_{total}-E_{warm\text{-}up}})]\},$$

where $E_{total}$ denotes the total number of training epochs; min_lr_ratio is the ratio of the minimum learning rate to $lr_{0}$, ensuring that the lr is not lower than the minimum rate. The continuity of the cosine function makes the learning rate decrease gradually and monotonically, avoiding the oscillation of the loss function. Figure 4 illustrates the learning rate variation governed by the WCA algorithm.

## 3. The Proposed Two-Stage Unet Framework

**Figure 5:**
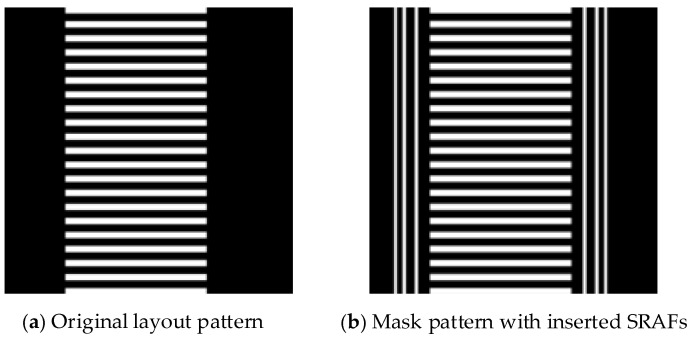
Example of the rectangular SRAFs inserted around the main pattern on the mask.

The proposed method requires rapid and accurate prediction of key parameters for inserting SRAFs within a specific layout region. Figure 5a shows the original layout pattern without SRAFs, where the main pattern includes line-space features. Figure 5b displays the mask pattern with six vertical SRAF bars inserted. Next, we will describe the proposed two-stage Unet framework in detail.

### 3.1. Unet1

**Figure 6:**
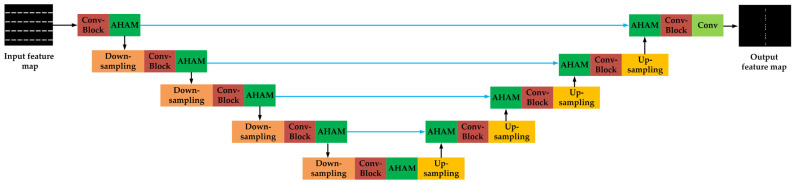
The architecture of the sub-network “Unet1”.

The detailed structure of the sub-network “Unet1” is shown in Figure 6, and its hyperparameters are shown in Appendix A. The Unet1 is constructed based on the Unet architecture. An AHAM module is introduced after the Conv-Block of each down-sampling stage. The fused map, which combines the up-sampling result and encoder feature map, is also fed into the AHAM module. Additionally, a convolution layer is added before the output layer to ensure that the output feature map has the same size as the input one.

For training purposes, the centroid coordinates of the training SRAF dataset need to be labeled in advance. These labels adopt the morphological dilation algorithm to expand the single centroid point into a square of a specified side length. The selection of the dilation side length affects the training effectiveness. An excessively small side length may cause the centroid labels to be recognized as noise, while a too large side length may induce overlaps between neighboring labels. To improve training efficiency and strengthen feature learning in the centroid region, the values of SRAFs’ centroid labels are increased (from 1 to 10). This adjustment encourages the network to extract the features in the centroid region, thereby enhancing prediction accuracy for SRAF centroids.

After the network training is complete, the feature map of initial centroid coordinates is obtained. Subsequently, the threshold segmentation algorithm is used to process the feature map [32], which is defined as follows:

(6) $$f(x_{i},y_{i})\left\{\begin{array}{l} 1\;f(x_{i},y_{i})>threshold\\ 0\;f(x_{i},y_{i})\leq threshold \end{array}\right.,$$

where $f(x_{i},y_{i})$ represents the value at row $x_{i}$ and column $y_{i}$ in the feature map. Subsequently, the connected component analysis-labeling algorithm [33] is used. It selects the regions exceeding the threshold as candidate regions, then calculates the centroids of the candidate regions. Based on the relationship between SRAFs and main patterns, additional centroid coordinates are added at the symmetric positions. Finally, adjacent centroid coordinates are merged to generate the final centroid coordinate map.

### 3.2. Unet2

**Figure 7:**
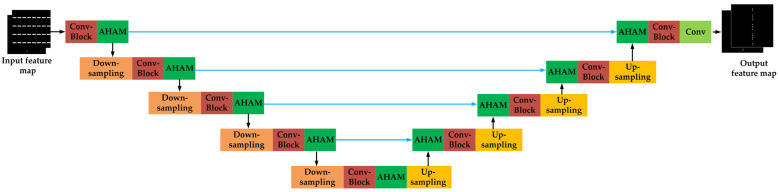
The architecture of the sub-network “Unet2”.

Figure 7 shows the structure of the sub-network “Unet2”, and Appendix B provides its hyperparameters. Similarly to Unet1, the AHAM module and convolution layer are introduced at the same position in Unet2. As shown in Figure 7, we adopt a dual-channel architecture design. At the input end, the first channel is the output map (centroid coordinate map) from “Unet1”, and the second channel is the original layout pattern. The higher weights are assigned to the first channel, thus making Unet2 predict the SRAF dimensional parameters around the regions of centroid coordinates. At the output end, the two channels correspond to the height and width parameters of SRAFs, respectively. After obtaining the outputs from Unet2, the regional maximum detection algorithm [34] is employed to extract the dimension information of SRAFs. Specifically, the algorithm identifies the maximum values in the output feature maps that correspond to the centroid regions, thereby estimating the width and height of SRAFs.

To generate ground-truth labels for SRAF parameters, the width and height values are first normalized to the range from 0 to 1, and subsequently assigned to the corresponding centroid coordinate regions. However, the lower normalized values of widths and heights may lead to issues such as gradient vanishing or neuron activation saturation when updating the network parameters, which may slow down the convergence rate and reduce the generalization performance. To solve this problem, higher weights are applied to the width and height labels, for example, by multiplying them by 1000.

After obtaining the output maps from both Unet1 and Unet2, we can determine the locations and sizes of all SRAF polygons. Subsequently, it is necessary to perform the mask rule check (MRC) for the inserted SRAFs. For the rectangular SRAFs in this work, MRC ensures that the line width meets the manufacturing constraints, avoiding the unexpected SRAF images printed on the wafer, and also eliminating the overlaps between SRAFs themselves or between SRAFs and main features. Finally, the MRC guarantees the manufacturability of the final mask pattern.

## 4. Simulation Results and Analysis

In the preliminary stage, we collect a total of 69 original layouts with a size of 1024×1024 pixels, where 60 of them are randomly selected as the training set and the remaining 9 layouts serve as the test set. The following simulations are conducted on a computer with an Intel (R) Xeon (R) E5-2620 v4 CPU and NVIDIA GeForce RTX 2080 Ti GPU. The proposed network is trained for 200 epochs, using the Adam optimizer. The maximum and minimum learning rates are 0.01 and 0.001, respectively. The warm-up stage of 10 epochs is applied to stabilize the initial training. The loss function based on the sum of squared due to errors (SSEs) is defined as follows:

(7) $$SSE=\sum_{x_{i}=1}^{m}\sum_{y_{i}=1}^{n}(G(x_{i},y_{i})-f(x_{i},y_{i}){)}^{2},$$

where $G(x_{i},y_{i})$ denotes the ground truth at the position $\left(x_{i},y_{i}\right)$ on the mask, $f(x_{i},y_{i})$ represents the corresponding predicted value.

### 4.1. Results

**Figure 8:**
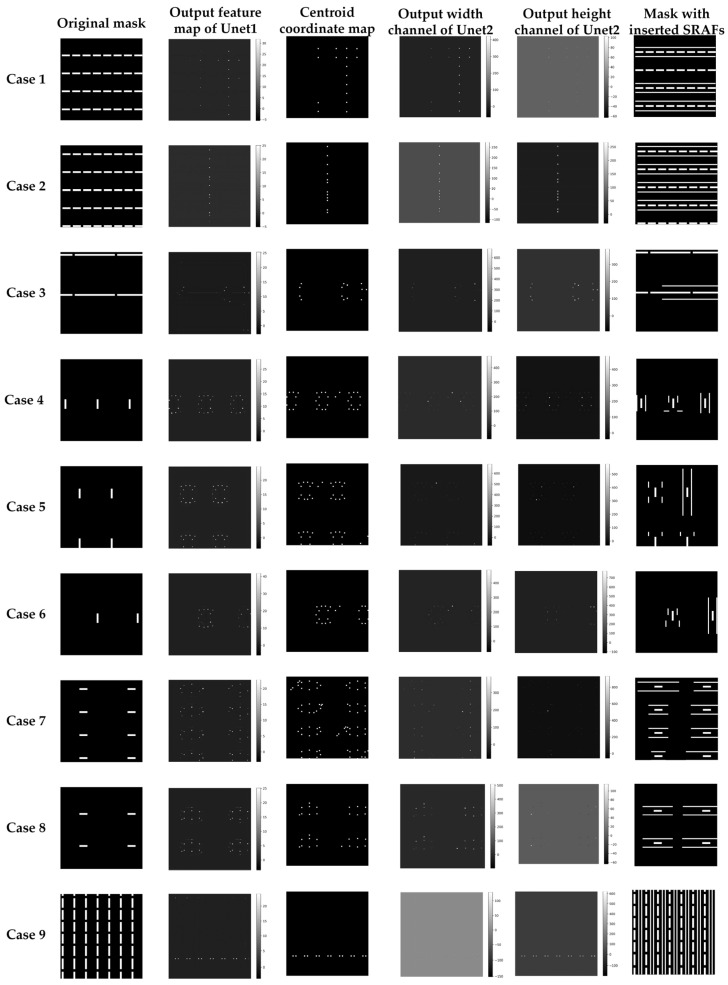
Simulation results for the proposed two-stage Unet method. From top to bottom, it shows 9 simulation cases with different layout patterns. From left to right, the columns illustrate the original mask pattern, the raw feature map output by Unet1, the post-processed feature map of Unet1, the feature map of the width channel output by Unet2, the feature map of the height channel outputted by Unet2, and the mask pattern including the predicted SRAFs, respectively.

The test set is fed into the well-trained network to evaluate prediction performance, and the simulation results of the test set are illustrated in Figure 8. From top to bottom, it shows 9 cases of simulations with different layout patterns. From left to right, the columns illustrate the original mask pattern, the raw feature map outputted by Unet1, the centroid coordinate map, which is obtained by processing the output feature map of Unet1, the feature map of the width channel output by Unet2, the feature map of height channel outputted by Unet2, and the mask pattern including the predicted SRAFs, respectively.

On closer inspection of Figure 8, column 2 exhibits significant differences in the value of the feature map output by Unet1 to different layouts. For example, the maximum value in the feature map for Case 6 is approximately 40, while that for Case 5 is about 20. Threshold segmentation and connected component analysis-labeling algorithm are applied to balance the output intensities across different cases. Further observation of columns 4 and 5 shows that the output maps of the height and width parameters successfully align with the centroid coordinate map in the post-processed map of Unet1. This demonstrates that the proposed method can accurately extract both the width and height for each SRAF in those regions. By comparing the results in columns 1 and 6, the proposed method can predict the appropriate number and sizes of SRAFs around the main pattern. Furthermore, the results of the 9 cases confirm that the proposed method inserts an adaptive SRAF for different types of mask patterns and that the stability and generalization ability of the proposed network.

**Figure 9:**
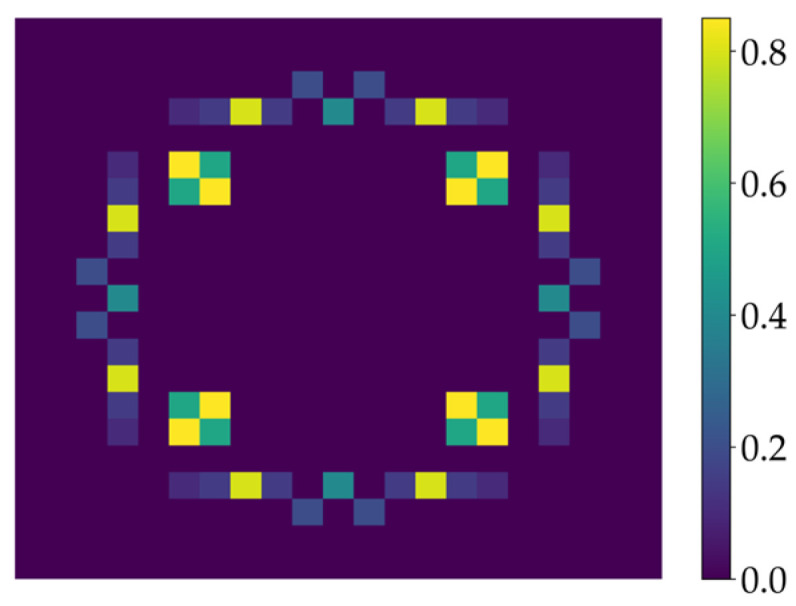
The pixelated source pattern used for simulating the aerial images of the lithography system.

To verify the effectiveness of the proposed method in improving lithography imaging quality, the Abbe’s method is used to simulate the aerial image of the mask patterns [35,36,37]. Then, the constant threshold resist model with a threshold of 0.19 is used as the resist model. In the following simulations, the illumination with 193 nm wavelength and XY polarization is used to calculate lithography images, and a pixelated source pattern is used for the lithography process, as illustrated in Figure 9. The numerical aperture (NA) of the projection optics is set to 1.2 with an immersion medium refractive index of 1.44. The demagnification factor of the projection system is 4.

Hereafter, the pattern error (PE) and edge placement error (EPE) are used to assess the imaging quality of the lithography system. PE is defined as the square of the Euclidean distance between the resist image and the target, while EPE represents the offset of the relative position between the resist imaging contour and the target pattern contour. In this paper, EPE is defined as the mean value of EPE at all pixel points along the target contour. PE and EPE are defined as follows:

(8) $$PE=\sum_{x_{i}=1}^{N}\sum_{y_{i}=1}^{N}{\left[Z_{T}(x_{i},y_{i})-\left.Z(x_{i},y_{i})\right]\right.}^{2},$$

(9) $$EPE=\frac{1}{L_{c}}\oint_{c}EPEdc,$$

where $Z_{T}(x_{i},y_{i})$ denotes the target pattern at the position $\left(x_{i},y_{i}\right)$, and $Z(x_{i},y_{i})$ represents the simulated resist image at the position $\left(x_{i},y_{i}\right)$. $L_{c}$ indicates the length of the target contour, and $\oint_{c}\cdot dc$ represents the line integral along the contour, and EPE represents the EPE value along the contour. A smaller EPE value corresponds to a higher image fidelity.

**Figure 10:**
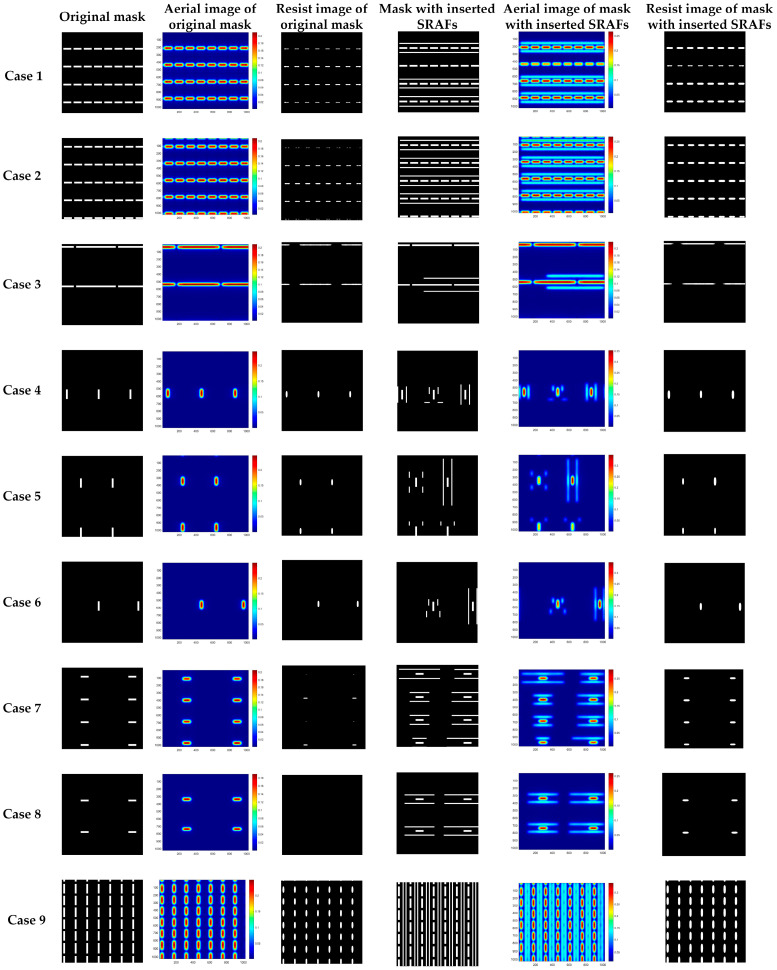
Lithography simulation results for the test set. From top to bottom, they show nine simulation cases with different layout patterns. From left to right, the columns show the original mask pattern, the aerial images of the original mask, the resist image of the original mask, the mask with inserted SRAFs (which were generated using the proposed method), the aerial images of the mask in column 4, and the resist image of the mask in column 4, respectively.

Figure 10 displays the lithography imaging simulation results for the test set. From top to bottom, it shows nine cases of simulations with different layout patterns. The first column shows the initial mask layouts without SRAFs. The second column and the third column show the aerial images and resist images corresponding to the masks in the first column. The fourth column shows the mask patterns with inserted SRAFs obtained by the proposed method. The fifth column and the sixth column show the aerial images and resist images corresponding to the masks in the fourth column. In these nine cases, although the intensities of the SRAFs could be observed, there are no residual images of SRAFs in the resist images after applying a threshold. Furthermore, compared with the resist images of original layouts, the inserted SRAFs will improve the imaging quality.

Table 1 lists the PEs and EPEs for all the resist images in Figure 10. As can be observed from Figure 10 and Table 1, the proposed method not only successfully inserts SRAFs at appropriate locations but also significantly reduces image errors. The PE and EPE values for all testing cases are reduced by different amounts. For PE, Case 2 demonstrates the most notable improvement, with PE reduced from 68,284 to 29,690, and Case 6 also displays a marked reduction in PE from 2296 to 1983. The mean PE across all test cases has decreased from 25,776.44 to 15,203.33. Similarly, the mean EPE value has declined from 5.8367 to 3.5283. Among them, Case 8 shows the largest decrease in EPE, dropping from 9.1667 to 3.2000, and Case 5 has the smallest decrease in EPE, dropping from 4.1903 to 3.5993. These results confirm that the proposed SRAF prediction method can effectively enhance the lithography image quality.

### 4.2. Ablation Study

In this section, an ablation study is designed to evaluate the contribution of each proposed strategy to the overall model performance. Accordingly, a total of four ablation experiments are conducted. The first one uses the traditional Unet framework to predict the SRAFs (“Unet” for short). The second one introduces the AHAM into the Unet (“Unet + AHAM” for short). The third one integrates the WCA algorithm into the Unet (“Unet + WCA” for short). The last one embeds both the WCA algorithm and AHAM into the Unet (“UNET + AHAM + WCA” for short). By comparing the results of those experiments, the contributions of individual improvement strategies, as well as their synergistic effects among them, can be clearly identified. The results of the ablation study are shown in Table 2, where the PEs and EPEs of lithography images for all cases are presented.

As shown in Table 1 and Table 2, for single improvement strategy, the “Unet + WCA” reduces the PE values by 6675, 4412, 200, 3873, 1497 and 2597 in Cases 1, 3, 4, 6, 7 and 8, respectively, while decreases the EPE values by 0.8691, 1.0750, 0.2364, 2.0088, 1.5594, 0.1876 in same cases, respectively. With the same number of training epochs, it outperforms “Unet” in SRAF insertion, indicating that the WCA algorithm accelerates the learning of key parameters and improves the training efficiency. Notably, in Cases 7 and 9, “Unet” fails to achieve effective SRAF insertion while “Unet + WCA” succeeds, demonstrating that the WCA algorithm enables SRAF insertion across diverse masks and enhances its generalization. The “Unet + AHAM” achieves lower PE and EPE values than both traditional “Unet” and “Unet + WCA” in most cases. The gain comes from the fact that the AHAM enhances the extraction ability of key features and the correlation information between main patterns and SRAF parameters.

For the joint improvement strategy, “Unet + AHAM + WCA” obtains the lowest PE and EPE values in Cases 1, 2, 6, 7, 8, and 9 than the other methods. This finding indicates that the synergy effect between the AHAM and the learning rate adjustment can further improve the prediction results. Although the PE and EPE values of “Unet + AHAM + WCA” are higher than the “Unet + AHAM” method in Cases 3, 4, and 5, its overall performance is still superior. Thus, these ablation experiments fully verify the effectiveness of the AHAM and WCA algorithm, as well as the benefit of jointly using both strategies.

### 4.3. Comparison with Other Methods

To further demonstrate the merits of the proposed method, some comparative experiments are conducted with several popular neural networks, including the fully convolutional network (FCN) [38], generative adversarial network (GAN) [39], conditional generative adversarial network (CGAN) [40], and attention Unet (AUnet) [41]. The results are listed in Table 3.

As shown in Table 3, the proposed method results in slightly higher PE and EPE values than the CGAN method in Cases 3, 4, 5, and 6. However, it achieves the optimal performance in all other scenarios, and the lowest average PE and EPE among all methods. In Cases 1, 2, 7, 8, and 9, the proposed method obtained PE values of 26,994, 29,690, 6699, 3072, and 43,694, respectively. These results demonstrate its superior performance compared to all other methods. These results exhibit the superiority of the predicted SRAFs obtained by the proposed method. In view of this superior performance, we believe the proposed method successfully addresses the inherent limitations of several popular neural networks. FCN lacks a mechanism to balance global and local features, which leads to insufficient SRAF prediction accuracy due to spatial information loss. GANs and CGANs suffer from the challenges of unstable training and mode collapse, which impair the generalization capability of SRAF predictions. Furthermore, AUnet relies on a single attention mechanism and cannot integrate global and local information in mask feature maps.

## 5. Conclusions

This paper proposed a novel deep-learning framework with a two-stage Unet architecture for rapidly predicting the key parameters of Manhattan SRAFs. The first stage of the proposed network was used to predict the centroid coordinates of SRAFs, while the second stage, with a dual-channel architecture, was designed to predict their dimensional parameters of SRAFs. The AHAM was introduced to dynamically adjust the weighting ratio between the global and local information in mask feature maps, thereby enhancing the network’s ability to capture the critical features and improving prediction accuracy. Additionally, the WCA algorithm was adopted, which significantly improved the stability and convergence rate of the training process. Experimental results demonstrated that the proposed method could accurately predict the SRAFs’ positions and sizes across multiple test cases, and the generated SRAFs can notably improve lithography imaging quality. The mean PE decreased from 25,776.44 to 15,203.33, while the mean EPE reduced from 5.8367 to 3.5283.

## Figures and Tables

**Table 1:** The comparison of PEs and EPEs before and after inserting SRAFs for all testing layouts.

		Case 1	Case 2	Case 3	Case 4	Case 5	Case 6	Case 7	Case 8	Case 9	Mean
Original Mask	PE	51,910	68,284	20,386	3545	4664	2296	15,401	8800	56,702	25,776.44
	EPE	6.7591	7.1576	4.9673	4.1903	4.1348	4.0709	7.9880	9.1667	4.0952	5.8367
Mask with inserted SRAFs	PE	26,994	29,690	17,237	3045	4416	1983	6699	3072	43,694	15,203.33
	EPE	3.5148	3.1122	4.2000	3.5993	3.9149	3.5160	4.4746	3.2000	3.1557	3.5283

**Table 2:** The comparison of PEs and EPEs obtained by the four ablation experiments based on all testing layouts.

	Unet		Unet + WCA		Unet + AHAM		Unet + AHAM + WCA	
	PE	EPE	PE	EPE	PE	EPE	PE	EPE
Case 1	51,910	6.7591	45,235	5.8900	36,612	4.7672	26,994	3.5148
Case 2	68,284	7.1577	68,284	7.1577	53,045	5.5603	29,690	3.1122
Case 3	20,386	4.9673	15,974	3.8923	10,264	2.5010	17,237	4.2000
Case 4	3227	3.8144	3027	3.5780	2872	3.3948	3045	3.5993
Case 5	4222	3.7429	4584	4.0638	4327	3.8360	4416	3.9149
Case 6	2135	3.7855	2146	3.8050	1989	3.5266	1983	3.5160
Case 7	15,234	7.9015	11,361	5.8926	8379	4.3460	6699	3.4746
Case 8	8800	9.1667	7303	7.6073	4415	4.5990	3072	3.2000
Case 9	46,427	3.3531	43,830	3.1655	43,797	3.1632	43,694	3.1557
Mean	24,513.89	5.6276	22,416.00	5.0058	18,411.11	3.9660	15,203.33	3.5208

**Table 3:** The comparison of PEs and EPEs obtained by the proposed method and some other comparative methods.

	Original Mask		FCN		GAN		CGAN		AUnet		Proposed Method	
	PE	EPE	PE	EPE	PE	EPE	PE	EPE	PE	EPE	PE	EPE
Case 1	51,910	6.7591	42,039	5.4738	51,910	6.7591	31,689	4.1262	29,176	3.7990	26,994	3.5148
Case 2	68,284	7.1577	65,274	6.8421	68,284	7.1577	50,376	5.2805	36,578	3.8342	29,690	3.1122
Case 3	20,386	4.9673	13,981	3.4067	20,386	4.9673	15,546	3.7880	20,386	4.9673	17,237	4.2000
Case 4	3545	4.1903	3147	3.7199	3545	4.1903	2786	3.2931	3497	4.1336	3045	3.5993
Case 5	4664	4.1348	4339	3.8466	4664	4.1348	4082	3.6188	4694	4.1613	4416	3.9149
Case 6	2296	4.0709	2140	3.7943	2296	4.0709	1866	3.3085	2291	4.0621	1983	3.5160
Case 7	15,401	7.9881	14,149	7.3387	15,121	7.8428	11,993	6.2204	14,628	7.5871	6699	3.4746
Case 8	8800	9.1667	7455	7.7656	8800	9.1667	5200	5.4167	7433	7.7427	3072	3.2000
Case 9	56,702	4.0952	52,311	3.7781	55,098	3.9793	49,385	3.5667	47,271	3.4141	43,694	3.1557
Mean	25,776.44	5.8367	22,759.44	5.1073	25,567.11	5.0877	19,213.67	4.2910	18,439.33	4.8557	15,203.33	3.5283

## Data Availability

The original contributions presented in the study are included in the article; further inquiries can be directed to the corresponding author.

## Appendix A

**Table A1:** Hyperparameters of the network structure of Unet1.

	Module	Input Feature Map Size	Output Feature Map Size	Convolution Parameters (Kernel/Stride/Padding)	Padding Mode
1	Conv_block	1 × 1024 × 1024	32 × 1024 × 1024	3 × 3/1/1	zeros
2	AHAM	32 × 1024 × 1024	32 × 1024 × 1024		
3	Down-sampling	32 × 1024 × 1024	32 × 512 × 512	3 × 3/2/1	zeros
4	Conv_block	32 × 512 × 512	64 × 512 × 512	3 × 3/1/1	zeros
5	AHAM	64 × 512 × 512	64 × 512 × 512		
6	Down-sampling	64 × 512 × 512	64 × 256 × 256	3 × 3/2/1	zeros
7	Conv_block	64 × 256 × 256	128 × 256 × 256	3 × 3/1/1	zeros
8	AHAM	128 × 256 × 256	128 × 256 × 256		
9	Down-sampling	128 × 256 × 256	128 × 128 × 128	3 × 3/2/1	zeros
10	Conv_block	128 × 128 × 128	256 × 128 × 128	3 × 3/1/1	zeros
11	AHAM	256 × 128 × 128	256 × 128 × 128		
12	Down-sampling	256 × 128 × 128	256 × 64 × 64	3 × 3/2/1	zeros
13	Conv_block	256 × 64 × 64	512 × 64 × 64	3 × 3/1/1	zeros
14	AHAM	512 × 64 × 64	512 × 64 × 64		
15	Up-samling	512 × 64 × 64	256 × 128 × 128	3 × 3/2/1 (ConvTranspose)	zeros
	fused with layer 11		512 × 128 × 128		
16	AHAM	512 × 128 × 128	512 × 128 × 128		
17	Conv_block	512 × 128 × 128	256 × 128 × 128	3 × 3/1/1	zeros
18	Up-samling	256 × 128 × 128	128 × 256 × 256	3 × 3/2/1 (ConvTranspose)	zeros
	fused with layer 8		256 × 256 × 256		
19	AHAM	256 × 256 × 256	256 × 256 × 256		
20	Conv_block	256 × 256 × 256	128 × 256 × 256	3 × 3/1/1	zeros
21	Up-samling	128 × 256 × 256	64 × 512 × 512	3 × 3/2/1 (ConvTranspose)	zeros
	fused with layer 5		128 × 512 × 512		
22	AHAM	128 × 512 × 512	128 × 512 × 512		
23	Conv_block	128 × 512 × 512	64 × 512 × 512	3 × 3/1/1	zeros
24	Up-samling	64 × 512 × 512	32 × 1024 × 1024	3 × 3/2/1 (ConvTranspose)	zeros
	fused with layer 2		64 × 1024 × 1024		
25	AHAM	64 × 1024 × 1024	64 × 1024 × 1024		
26	Conv_block	64 × 1024 × 1024	32 × 1024 × 1024	3 × 3/1/1	zeros
27	Conv	32 × 1024 × 1024	1 × 1024 × 1024	3 × 3/1/1	zeros

## Appendix B

**Table A2:** Hyperparameters of the network structure of Unet2.

	Module	Input Feature Map Size	Output Feature Map Size	Convolution Parameters (Kernel/Stride/Padding)	Padding Mode
1	Conv_block	2 × 1024 × 1024	32 × 1024 × 1024	3 × 3/1/1	zeros
2	AHAM	32 × 1024 × 1024	32 × 1024 × 1024		
3	Down-sampling	32 × 1024 × 1024	32 × 512 × 512	3 × 3/2/1	zeros
4	Conv_block	32 × 512 × 512	64 × 512 × 512	3 × 3/1/1	zeros
5	AHAM	64 × 512 × 512	64 × 512 × 512		
6	Down-sampling	64 × 512 × 512	64 × 256 × 256	3 × 3/2/1	zeros
7	Conv_block	64 × 256 × 256	128 × 256 × 256	3 × 3/1/1	zeros
8	AHAM	128 × 256 × 256	128 × 256 × 256		
9	Down-sampling	128 × 256 × 256	128 × 128 × 128	3 × 3/2/1	zeros
10	Conv_block	128 × 128 × 128	256 × 128 × 128	3 × 3/1/1	zeros
11	AHAM	256 × 128 × 128	256 × 128 × 128		
12	Down-sampling	256 × 128 × 128	256 × 64 × 64	3 × 3/2/1	zeros
13	Conv_block	256 × 64 × 64	512 × 64 × 64	3 × 3/1/1	zeros
14	AHAM	512 × 64 × 64	512 × 64 × 64		
15	Up-samling	512 × 64 × 64	256 × 128 × 128	3 × 3/2/1 (ConvTranspose)	zeros
	fused with layer 11		512 × 128 × 128		
16	AHAM	512 × 128 × 128	512 × 128 × 128		
17	Conv_block	512 × 128 × 128	256 × 128 × 128	3 × 3/1/1	zeros
18	Up-samling	256 × 128 × 128	128 × 256 × 256	3 × 3/2/1 (ConvTranspose)	zeros
	fused with layer 8		256 × 256 × 256		
19	AHAM	256 × 256 × 256	256 × 256 × 256		
20	Conv_block	256 × 256 × 256	128 × 256 × 256	3 × 3/1/1	zeros
21	Up-samling	128 × 256 × 256	64 × 512 × 512	3 × 3/2/1 (ConvTranspose)	zeros
	fused with layer 5		128 × 512 × 512		
22	AHAM	128 × 512 × 512	128 × 512 × 512		
23	Conv_block	128 × 512 × 512	64 × 512 × 512	3 × 3/1/1	zeros
24	Up-samling	64 × 512 × 512	32 × 1024 × 1024	3 × 3/2/1 (ConvTranspose)	zeros
	fused with layer 2		64 × 1024 × 1024		
25	AHAM	64 × 1024 × 1024	64 × 1024 × 1024		
26	Conv_block	64 × 1024 × 1024	32 × 1024 × 1024	3 × 3/1/1	zeros
27	Conv	32 × 1024 × 1024	2 × 1024 × 1024	3 × 3/1/1	zeros
